# Demographic history, genetic structure and gene flow in a steppe-associated raptor species

**DOI:** 10.1186/1471-2148-11-333

**Published:** 2011-11-17

**Authors:** Jesus T Garcia, Fernando Alda, Julien Terraube, François Mougeot, Audrey Sternalski, Vincent Bretagnolle, Beatriz Arroyo

**Affiliations:** 1 Instituto de Investigación en Recursos Cinegéticos (IREC) (CSIC-UCLM-JCCM). Ronda de Toledo s/n, E-13005 Ciudad Real, Spain; 2 Natural Research Ltd, Banchory Business Centre, Burn O'Bennie Road, Banchory, AB31 5ZU, UK; 3 Estación Experimental de Zonas Áridas (EEZA-CSIC), Ctra. de Sacramento s/n, La Cañada de San Urbano, 04120 Almería, Spain; 4 CEBC-CNRS, UPR1934, 79360 Beauvoir sur Niort, France

## Abstract

**Background:**

Environmental preferences and past climatic changes may determine the length of time during which a species range has contracted or expanded from refugia, thereby influencing levels of genetic diversification. Connectivity among populations of steppe-associated taxa might have been maximal during the long glacial periods, and interrupted only during the shorter interglacial phases, potentially resulting in low levels of genetic differentiation among populations. We investigated this hypothesis by exploring patterns of genetic diversity, past demography and gene flow in a raptor species characteristic of steppes, the Montagu's harrier (*Circus pygargus*), using mitochondrial DNA data from 13 breeding populations and two wintering populations.

**Results:**

Consistent with our hypothesis, Montagu's harrier has relatively low genetic variation at the mitochondrial DNA. The highest levels of genetic diversity were found in coastal Spain, France and central Asia. These areas, which were open landscapes during the Holocene, may have acted as refugia when most of the European continent was covered by forests. We found significant genetic differentiation between two population groups, at the SW and NE parts of the species' range. Two events of past population growth were detected, and occurred ca. 7500-5500 and ca. 3500-1000 years BP in the SW and NE part of the range respectively. These events were likely associated with vegetation shifts caused by climate and human-induced changes during the Holocene.

**Conclusions:**

The relative genetic homogeneity observed across populations of this steppe raptor may be explained by a short isolation time, relatively recent population expansions and a relaxed philopatry. We highlight the importance of considering the consequence of isolation and colonization processes in order to better understand the evolutionary history of steppe species.

## Background

Contemporary patterns of genetic diversity and population structure reflect not only current patterns of genetic exchange but also past dispersal processes and levels of gene flow among populations during historical climatic events [1,2]. In many species inhabiting temperate zones, climate-vegetation feedbacks during the Pleistocene caused range contractions to lower latitudes followed by range expansions during interglacial periods [2,3], which in turn promoted much of the diversification observed today. However, not all species responded similarly to these past climatic events. Species-specific responses to these changes are the result of a complex interplay between the behavioral, physiological and ecological characteristics of the species, including their biogeographic origin, habitat preferences and dispersal capabilities [4]. Climatic conditions prevailing at different time periods strongly influenced the extent of each habitat type in the past, which should in turn influence the length of time during which a species underwent isolation or range expansion and, consequently, the opportunities for genetic diversification [3,5]. For example, species inhabiting arctic or boreal areas seem to have experienced range expansions during the long glacial periods, but remained isolated during the short interglacial ones [6-8], leading to a pattern of contemporary genetic structure different from that of species inhabiting temperate areas. The phylogeography of temperate and arctic species is rather well studied. In contrast, the phylogeography of steppe species, which are biogeographically in-between the temperate and arctic-boreal fauna, remains scarcely known [4,9]. During the glacial periods, many of these species were widely distributed throughout the periglacial steppes of the northern hemisphere. For these steppe species, gene flow at large geographic scales might have been interrupted by the postglacial retreat and reduction of steppe vegetation during the short interglacial phases. Therefore, the amount of time spent in isolation, and the resulting genetic differentiation, should be smaller for steppe species as compared with temperate ones. The distribution of steppe species during the glacial periods is well documented by fossil evidence [10-12], but the genetic evidence is still relatively poor [9,13-15]. In addition, steppe-like ecosystems (including natural and agricultural landscapes) are probably amongst the most altered habitats nowadays, due to human pressure and rapid changes in land use [16]. Consequently, the abundance and distribution range of many steppe/farmland species has greatly declined in recent years [17-22].

**Figure 1:**
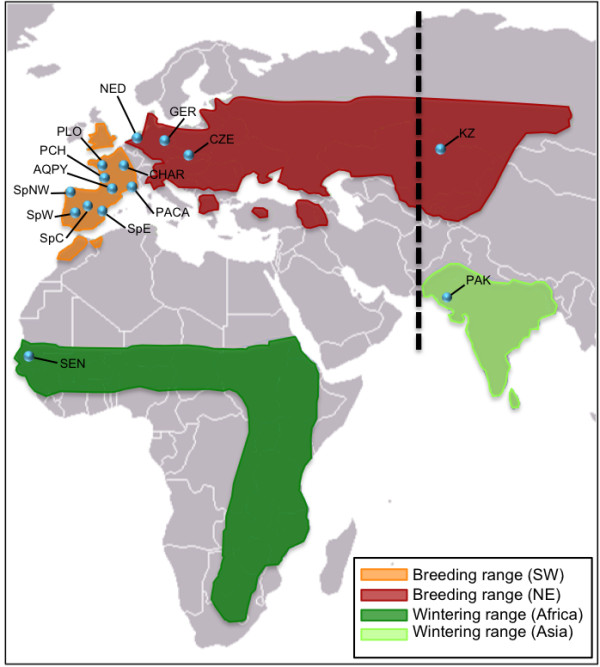
**Map showing the geographic range of breeding populations of Montagu's harrier (redrawn from**[86]) **and sampling localities used in this study**. Orange and red colors correspond to breeding grounds; the large lump in the breeding distribution in the old USSR reflects a lack of detailed knowledge on their distribution rather than a continuous range. The figure shows the separations between the two main distribution areas within the range. Green colors correspond to wintering grounds. Locality codes are as indicated in Table 1. The doted black line shows the location of the intercontinental migratory divide proposed by Moreau [27] to split the breeding populations wintering in the Indian sub-continent from those wintering in Africa

The Montagu's harrier *Circus pygargus *is perhaps the best example of raptor species specialized in such steppe habitats, which has also adapted well to farmland habitats [23,24]. This species is widely but patchily distributed across the Palearctic region and undertakes long-distance migrations [25] (Figure 1). Migration studies based on satellite tracking and ringing recoveries show that European and Asian breeding populations follow different migratory pathways and overwinter in different continents (Figure 1). Central Asian populations, where it is accepted that the stronghold of the species occurs (ca. 25,000-30,000 pairs [26]), migrate along an eastern route and overwinter in the Indian subcontinent (Pakistan and India [27]). European populations migrate through the Mediterranean peninsulas and overwinter in the Sahel belt from western to eastern Africa [28-30]. To date, no specific study has evaluated the genetic structure and gene flow among populations of this species or closely related ones.

We used molecular analyses to evaluate levels of genetic diversity and connectivity among Montagu's harrier breeding populations. We collected genetic material from across the whole breeding range (Spain to Kazakhstan) and from two overwintering populations (located in western Africa and in the Indian sub-continent). With this information, we analyzed historical demographic patterns, in order to better understand how past climatic dynamics may have affected the species and shaped its current distribution and population genetic structuring. Specifically, our aims were: first, to evaluate the genetic diversity of Montagu's harrier populations and analyze different scenarios that may explain the observed genetic structure (according to physical barriers, overwintering areas or current breeding distribution); second, to investigate past demographic changes in the species, and patterns of gene flow between genetically structured populations, looking for evidence of asymmetrical exchanges between these. Finally, we discuss the implications of these results for the understanding of the patterns shaping the phylogeographic structure of the fauna associated with steppe ecosystems.

## Results

We sequenced a total of 1063 bp of t-RNA Trp and ND2 and 714 bp of COI genes from all samples (n = 284), which were collapsed into 36 and 13 haplotypes, respectively (Additional file 1). The concatenated data set (1777 bp) yielded 51 different haplotypes defined by 35 polymorphic sites. Overall, gene and nucleotide diversities were Hd = 0.663 (SD, 0.032) and π = 0.0008 (SD, 0.00007), respectively (Table 1). Most of the observed genetic variability was in the form of single nucleotide substitutions. The mean number of pairwise nucleotide differences for all samples (concatenated data set) was 1.47 (SD, 0.94).

**Table 1:** Sample sizes and genetic characteristics.

Country	Region	**Abrev**.	n	nH	Hd (± SD)	π (± SD)	*D*	*F_S_*
*Breeding range*								
Spain	Extremadura	SpW	38	8	0.3798 ± 0.100	0.000469 ± 0.00038	-2.26*	-3.28*
Spain	Toledo, Ciudad Real	SpC	12	4	0.5606 ± 0.150	0.000639 ± 0.00050	-1.53*	0.62
Spain	Castellón	SpE	22	6	0.6797 ± 0.095	0.000672 ± 0.00041	-1.19	-0.96
Spain	Galicia	SpNW	20	8	0.7421 ± 0.096	0.000758 ± 0.00055	-1.82*	-3.71*
France	Provence-Alps-Cote Azur	PACA	19	7	0.8246 ± 0.064	0.000987 ± 0.00067	-0.92	-0.24
France	Pays Loire	PLO	6	4	0.8667 ± 0.125	0.001651 ± 0.00116	-0.49	1.36
France	Poitou-Charente	PCH	51	13	0.6894 ± 0.070	0.000954 ± 0.00063	-1.55*	-3.17
France	Champagne-Ardenne	CHAR	21	8	0.6762 ± 0.111	0.000820 ± 0.00058	-1.84*	-0.36
France	Aquitaine-Pyrenees	AQPY	10	5	0.7556 ± 0.129	0.000538 ± 0.00045	-1.11	-0.33
The Netherlands	Oldenzaal	NED	12	4	0.4545 ± 0.170	0.000963 ± 0.00068	-0.84	0.32
Germany	Lower Saxony	GER	6	2	0.3333 ± 0.215	0.000188 ± 0.00025	-0.93	-0.00
Czech Republic	Vysočyna	CZE	13	4	0.6026 ± 0.130	0.000909 ± 0.00065	0.45	0.32
Kazakhstan	Naurzum, Kostanay	KZ	32	8	0.6875 ± 0.077	0.000845 ± 0.00058	-0.60	-0.61
*Wintering areasWintering areas*								
Senegal	Nianing	SEN	10	2	0.3556 ± 0.155	0.000200 ± 0.00024		
Pakistan	Hyderabad	PAK	12	8	0.9242 ± 0.057	0.001808 ± 0.00113		
*Geographic originGeographic origin*								
SW							-2.27*	-28.5*
NE							-1.18	-10.2*
*Pooled*			*284*	*51*	*0.663 ± 0.032*	*0.000826 ± 0.00007*		

Summary of the number of samples (n), number of haplotypes (nH), haplotype diversity (Hd) and nucleotide diversity (π) for the concatenated data set in each sampling locality. Tajima's (*D*), Fu's (*F_S_*) measure departure of the data from neutrality. The significance (at the *P *= 0.05 level) of these statistics is indicated with an asterisk (*) for each breeding population and for the SW and NE demes (see text). SD = standard deviation.

French populations showed the highest nucleotide and haplotype diversities when considering each gene partition separately (ND2 and COI, results not shown) or the concatenated data set (Table 1). Haplotype diversities were also high in the coastal regions of Spain (Galicia and Castellón). The lowest haplotype diversities were found in populations from Germany and Netherlands (Table 1). Regarding the two wintering sites, levels of genetic diversity were greater in Pakistan than in Senegal (Table 1).

### Genetic structure

**Figure 2:**
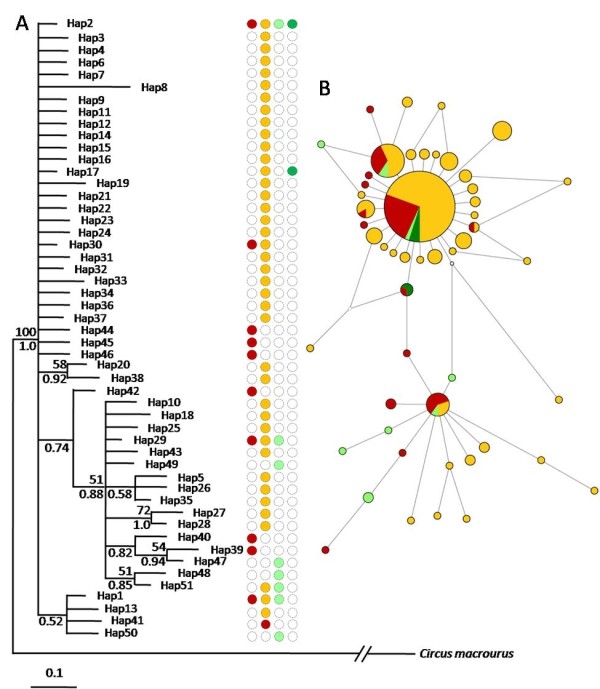
**Phylogenetic relationships among mitochondrial DNA**. A) Maximum-likelihood (ML) tree of *Circus pygargus *based on mtDNA sequences. Numbers above branches indicate ML bootstrap values (1000 replicates) and numbers below branches indicate BI posterior probabilities. Only bipartitions with bootstrap or posterior probability values above 50 and 0.5, respectively, are shown. B) Median-joining haplotype network based on the mtDNA concatenated data set. Circle size is proportional to haplotype frequency and connecting lines are proportional to mutation steps between haplotypes. White circles represent hypothetical intermediate or un-sampled haplotypes. Each color refers to one geographic area (orange: SW populations; red: NE populations; dark green: Senegal; light green: Pakistan).

The two phylogenetic methods used (Maximum likelihood and Bayesian inference) were largely consistent in the (lack of) relationships recovered. Despite the broad geographic sampling (Figure 1), there was little phylogenetic structure and low branch support (Figure 2). The haplotype network revealed no major branching events (Figure 2), although two groups of haplotypes could be differentiated. The first group was distributed around haplotype Hap2, which was observed in 57% of individuals and had a widespread geographical distribution. The other haplotypes were generally site-specific and occurred at low frequencies. The second group consisted mostly of rare haplotypes from all geographic regions except Senegal. In general, haplotypes specific to certain geographic regions did not form monophyletic groups, but appeared to be randomly distributed across the network (Figure 2).

Differences among Montagu's harrier populations accounted for a significant 5.15% of total molecular variance (Table 2). Hierarchical analyses testing for differences between populations in relation to their geographic origin showed low but significant differentiation among groups of populations located in the south-western (SW) vs. north-eastern (NE) parts of the breeding range (scenario 1), with low levels of genetic variation among populations within each group. In contrast, the structure scenarios based on geographic barriers (scenario 2) or based on an intercontinental migratory divide (scenario 3) revealed no significant among-group differences (Table 2). The Φ_CT _estimate obtained for scenario 3 was similar to that obtained for scenario 1, and close to significance (Table 2). This suggested some structuring in relation to the migratory divide, although the higher and significant value of Φ_ST _indicated larger heterogeneity among populations within groups than in scenario 1. Including samples from wintering areas might affect, to some extent, these results, but the exclusion of these wintering samples also entails the loss of important genetic information. We repeated these analyses without including the samples from the two overwinter sites, and results were qualitatively the same.

**Table 2:** AMOVA summary.

**Concatenated data**					
			**% of total variance (p-value)**		
**Scenario**	**Grouping**	**Populations in group**	**Between group Φ_CT_**	**Between population within group Φ_ST_**	**Within population Φ_IS_**
			
1. Geographic distribution	Southwestern *vs*. Northeastern	(SpW, SpC, SpNW, SpE, PACA, PLO, PCH, AQPY, CHAR) vs. (NED, GER, CZE, KZ, PAK)	**4.23 (0.0009)**	**3.21 (0.011)**	**92.56 (0.000)**
2. Geographic barriers	Spain *vs*. Western Europe *vs*. Eastern Europe *vs*. central Asian	(SpW, SpC, SpNW, SpE) vs. (PACA, PLO, PCH, AQPY, CHAR, NED, GER) vs. (CZE) vs. (KZ, PAK)	2.48 (0.114)	**3.42 (0.007)**	**94.10 (0.000)**
3. Migratory divide	Africa vs. India	(SpW, SpC, SpNW, SpE, PACA, PLO, PCH, AQPY, CHAR, NED, GER, CZE, SEN) vs. (KZ, PAK)	4.55 (0.087)	**3.66 (0.0009)**	**91.78 (0.000)**
No grouping				**5.15 (0.000)**	**94.85 (0.000)**

Results of AMOVA (concatenated data, Tamura and Nei; gamma = 0.2487) testing for differences between Montagu's harrier populations and geographic areas. The analysis partitions out total molecular variance into different components, and statistical significance is obtained by randomization after 5000 permutations. Significant differences between groups are shown in bold.

Overall, 27 out of 105 pairwise Φ_ST _comparisons between populations were significant. Comparisons involving Pakistan were significant in all but four cases (Table 3). Differences were smallest between Pakistan and Czech Republic (Φ_ST _= 0.037) and largest between Pakistan and western Spain (PAK-SpW: Φ_ST _= 0.324). In contrast, comparisons involving Senegal were significant only with central France (PLO) and Pakistan (SEN-PLO: Φ_ST _= 0.159, and SEN-PAK: Φ_ST _= 0.224). Considering only breeding populations, eastern Spain (SpE) showed the highest number of significant pairwise comparisons (seven out of 12), while the largest differentiation among populations was found between western Spain (SpW) and central France (PLO) (SpW-PLO: Φ_ST _= 0.216). Finally, the correlation between population pairwise Φ_ST _values and their geographical distances was not significant (Mantel test, r = -0.06, p = 0.70).

**Table 3:** Between-population genetic differentiation in *Circus pygargus*.

	SpW	SpC	SpE	SpNW	PACA	PLO	PCH	CHAR	AQPY	NED	GER	CZE	KZ	SEN	PAK
SpW	--		*			*		*				*	*		***
SpC	0.0115	--													*
SpE	0.0346	0.0066	--		*	**	*	***		*		**	*		***
SpNW	0.0022	-0.0098	0.0157	--		*									*
PACA	0.0353	0.0144	0.0386	-0.0056	--										*
PLO	0.2161	0.1091	0.1646	0.1312	0.0897	--							*	*	
PCH	0.0156	-0.0050	0.0395	-0.0009	0.0137	0.0667	--								***
CHAR	0.0314	0.0079	0.0491	0.0280	0.0268	0.0781	0.0148	--				*			**
AQPY	0.0233	0.0330	0.0387	0.0305	0.0132	0.1028	0.0236	-0.0270	--			*			***
NED	0.0436	-0.0051	0.0657	0.0181	0.0143	0.0648	-0.0049	0.0279	0.0537	--					
GER	-0.0680	-0.0239	-0.0470	-0.0816	-0.0629	0.1068	-0.0453	-0.0308	-0.0000	0.0000	--				
CZE	0.1290	0.0445	0.1278	0.0546	0.0286	0.0786	0.0209	0.0891	0.1350	-0.0183	0.0787	--			
KZ	0.0386	0.0131	0.0559	-0.0030	-0.0077	0.1209	0.0051	0.0518	0.0651	0.0018	-0.0399	-0.0037	--		*
SEN	-0.0030	-0.0044	0.0195	-0.0177	0.0109	0.1596	-0.0182	0.0052	0.0635	0.0035	0.0585	0.0656	0.0087	--	*
PAK	0.3241	0.1891	0.2781	0.2068	0.1541	0.1246	0.1928	0.2415	0.2503	0.0660	0.1826	0.0370	0.1451	0.2240	--

Below the diagonal, pairwise Φ_ST _values (based on Tamura and Nei distances between haplotypes; gamma = 0.248). Above the diagonal, p-values for significant Φ_ST _values obtained after 5000 permutations (* p < 0.05; ** p < 0.01, *** p < 0.001).

### Demographic analyses

The whole breeding population exhibited significantly negative values for both Tajima's *D *(*D *= -2.28, p = 0.0023) and Fu's *F_S _*(*F_S _*= -68.94, p < 0.001), suggesting that the overall population size has fluctuated in the past. The majority of *D *and *F_S _*values were negative for most of the studied populations, but Fu's *F_S _*rejected neutrality (p < 0.02) in only two breeding populations, whereas Tajima's *D *rejected neutrality (p < 0.05) in five populations (Table 1). We further considered the two groups of populations (from the SW and NE regions) whose genetic structure differed according to AMOVA analysis (see above). Each group of populations also showed negative *D *and *F_S _*values that were all significant except for the Tajima's *D *of the NE group (Table 1).

**Figure 3:**
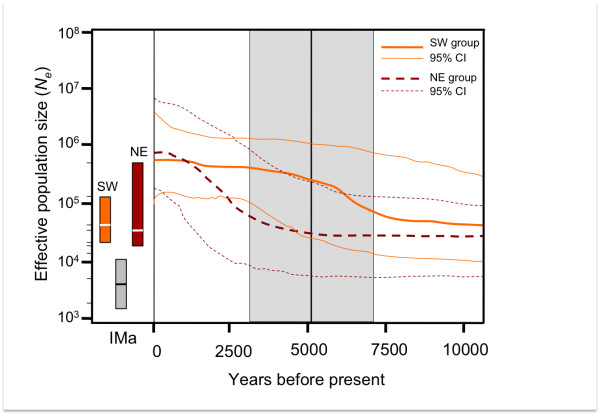
**Bayesian skyline plot generated with BEAST v1.5.4. and effective population size (*Ne*) and time estimates under IMa**. The solid red lines are the median and 95% confidence intervals (CI) estimate for SW populations while dotted blue lines for NE populations. The shaded box in the centre of the figure represents the mean and 95%CI for the time of population splitting estimated by IMa for SW and NE groups of Montagu's harriers. Boxes represent 95% CI for the *N_e _*estimated by IMa with SW and NE groups and the ancestral population in grey. Lines inside the boxes represent the peak of the marginal distribution values.

The effective population sizes and demographic trends estimated by the Bayesian Skyline Plot (BSP) analysis indicated recent population size increases in both regions (SW and NE). However, the overall increase was much less marked for the SW populations, which showed a roughly linear increase during the second part of the Holocene. Based on a range of mutation rates from 0.02 to 0.055 s/s/Myr, population growth started at approximately 7500 years before present (BP) in SW and 4000 years BP in NE (Figure 3). The time to the most recent common ancestor (TMRCA) was estimated at 21,000 (40,000-6000 95% highest posterior density, HPD) and 35,000 years BP (61,000-13,000 95% HPD) for SW and NE groups, respectively. Independent runs of IMa gave similar results, and plots of parameter trends indicated sufficient mixing among chains. The estimated effective population size for the SW region (peak θ*_SW_*= 75.18, 90% HPD = 41.4 - 123.1; Figure 3) was similar to that of the NE region (peak θ*_NE _*at 63.45, 90% HPD = 16.5 - 397.0; Figure 3), despite larger HPD in NE than in SW group. Both descendant populations had effective population sizes that were over one order of magnitude larger than the ancestral population (θ*_A_*), which peaked at 10.28 (90% HPD = 2.82 - 17.57). Based on these values of θ, both populations appeared to have grown substantially following divergence. The average estimate for the scaled splitting time was *t *= 0.37 (90% HPD = 0.22 - 0.50; Figure 3), suggesting that the NE and SW groups of populations started to diverge about 5200 years BP (7100 - 3100 years BP considering the range of mutation rates used). The gene flow estimate from SW into NE was low (*m *= 0.66, 90% HPD = 0.0035 - 4.12), and close to null from NE into SW (*m *= 0.0035, 90% HPD = 0.0035 - 2.88). Conversion of these values of *m *resulted in an estimated number of migrants of approximately 0.004 females per generation from NE to SW populations (one female every 250 generations), and 22 females per generation from SW to NE populations. However, the associated error to these estimates was large (90% HPD_SW _= 0.1 - 108; 90% HPD_NE _= 0.1 - 130 female migrants per generation).

## Discussion

### Genetic diversity of Montagu's harrier

Overall, we found little mitochondrial DNA variation among populations of Montagu's harrier throughout the breeding range. Although variability was low, we nevertheless found significant differentiation between southwestern and northeastern populations. In contrast to the low among-population variability, our study revealed a relatively high degree of genetic diversity at the mtDNA for this species as compared with those found in other sympatric raptor species. These other raptors often show much less polymorphism, even when using genetic markers that are more variable than the mitochondrial control region (examples from vultures in [31]). Some of these studies have associated these low levels of genetic diversity with recent decreases in population sizes, like for instance in the Spanish imperial eagle *Aquila adalberti *[32], Red kite *Milvus milvus *[33], Bonelli's eagle *Hieraaetus fasciatus *[34], White-tailed eagle *Haliaeetus albicilla *[35], and Bearded vulture *Gypaetus barbatus *[36]. The mtDNA diversity of the Montagu's harrier is not consistent with a recent population decline, unlike in these other studied raptor species. However, genetic diversity is rarely homogeneously distributed within a species range, because it reflects the long-term effects of historical events [2]. Despite relatively small sample sizes for some localities, we were able to identify two strongholds of genetic diversity for the Montagu's harrier, one in the western part of the range (France and coastal Spain), and another in central Asia. This coincides with the distribution of open vegetation in the mid Holocene, which was restricted to the Iberian Peninsula and coastal areas of France and to central Asia [37-39] suggesting that these areas may have acted as refuges for the species at a time when most of Europe was covered by dense forest [40]. Fossil records confirm the presence of Montagu's harriers in the western region from the Pleistocene through the Holocene [12]. However, our lack of sampling locations in East Asia prevents us from pinpointing primary areas of Montagu's harrier diversity in this region. Additional sampling would help to establish if the diversity found in Kazakhstan reflects higher levels of diversity in the Asian steppes, as suggested by the high diversity found in the wintering grounds of Asian breeding populations (PAK) compared to their west European counterparts (SEN).

The haplotype network consisted of two distinct, but closely related, lineages (Figure 2) that could also be indicative of two refugia or nuclei from where the species expanded. The existence in Eurasia of a western and an eastern or central refuge area has been proposed for other bird species [31,35,41,42], and the highest values of genetic diversity observed in France and in Kazakhstan would be consistent with such scenario. However, we found no relationship between lineages and their geographic origin (Figure 2). This could be a consequence of the low divergence between the two haplotype groups, suggesting a short time of isolation for these populations. This would be expected for a steppe bird whose habitat was potentially reduced during a short period within the present interglacial period, rather than during the longer glacial periods, as postulated for temperate species [4]. During the Pleistocene, the duration of the steppe-favorable period would have allowed harriers to fully expand their range, leading to a high connectivity between different demes. This could have erased any phylogeographic signal, as is commonly observed in species characterized by a high dispersal capability and exploiting a wide ecological niche [35].

### Population structure and phylogeography

In many animal species, the patterns of genetic differentiation and gene flow are highly influenced by the geographical characteristics of the places they inhabit and by their migratory behavior. However, in the Montagu's harrier, neither the geographical barriers (mountain ranges) nor the intercontinental migratory divide (populations wintering in Africa or in the Indian sub-continent) represented significant barriers against gene flow. Interestingly, the levels of genetic differentiation between populations were unrelated to the geographical distances separating these populations. This could be explained by the long-distance migration behavior of the species. Western European breeding birds (Spain, France) winter primarily in west Africa (Senegal, Mauritania and Mali [30,43]), while central and eastern-European harriers (the Netherlands, Czech Republic and Germany in this study) winter further east (Niger, Nigeria and Chad [29]). Montagu's harriers may perform winter movements following outbreaks of locusts and grasshoppers (their main food in winter) [29]. Additionally, wind conditions may influence the migratory routes, particularly in spring [44]. Some individuals might thus migrate through a different route upon their return (spring) migration, and finally disperse and breed far away from their natal place. This could explain the lack of significant genetic structure between populations *a priori *assigned to different overwintering areas. This hypothesis of different migration routes between spring and autumn is supported by ring recoveries [28] and counts of migratory birds in the central Mediterranean [45], although satellite telemetry-based studies (conducted on a limited number of individuals) indicate that most birds follow the same route in spring and autumn migrations [46]. While the correlation between genetic and geographical distances for all populations was non-significant, the AMOVA test gave statistical evidence for a differentiation between the SW and NE populations (although only 5% of the genetic variation was explained by this partition). In other words, location of the breeding areas may be important when explaining the genetic structure of populations at a broad scale, whereas geographical distance between populations is not. These observations, together with the low number of significant comparisons between pairs of populations, points to dispersal as a major factor preventing genetic differentiation within these two regions of the breeding range of Montagu's harriers. Such a relaxed philopatric behavior has indeed been described for the species [47].

### Demographic history

Our results strongly support a recent population expansion as an important cause of the relative homogeneity across populations. Such expansion is indicated by the shallow phylogenetic tree and by the star-like haplotype network (Figure 2). The genetic signature observed in the two main Montagu's harrier groups of populations (SW vs. NE) is consistent with the occurrence of postglacial demographic expansions during the second half of the Holocene, as evidenced by the BSP analyses. These revealed two events of population growth that occurred first in the SW (ca. 7500-5500 years BP), and later in the NE part of the range (ca. 3500 to 1000 years BP; Figure 3). The effective population size increases also appeared more pronounced in the NE than in the SW populations (Figure 3). Furthermore, these two groups of populations diverged around 5200 years ago, between the two waves of population growth. This pattern may be explained by regional differences in the impact of both climate changes and human activity on vegetation during the Holocene. Ample evidence support that during the last glacial maximum (37,000-16,000 years BP), the tundra-steppe vegetation was widespread from France to the Bering strait (e.g. [48]). Therefore, according to the preference of Montagu's harrier for open steppe-like landscapes, the species would have been widely distributed throughout Eurasia. Then, during the following interglacial period at the beginning of the Holocene, about 10,000 years ago, this vast system disappeared almost completely as a consequence of the quick expansion of temperate forests (in Europe) and taigas (in Asia) from their ice-Age refugia [49]. This probably led to strong range contraction and population declines in steppe-associated communities. Unfortunately, based on our data set, coalescent times go back only 10,000 years, therefore changes in population size before that date (e.g. after the last glacial maximum ca.18,000 to 10,000 years BP) cannot be inferred.

Although this particular issue has received little attention so far (phylogeographic studies of steppe species are still scarce), our data are consistent with the recent idea of interglacial refugia, which proposes that, in addition to the traditional high-latitude refugia of boreal species, cryptic refugia might have existed in other areas in the south during the interglacials [4,9]. In fact, climate reconstructions based on pollen records have shown that, during the Holocene, the climate was neither stable nor uniform across Eurasia [50-52]. Therefore, the occurrence of these cryptic refugia, and consequently the severity of climate change impacts on species, might have been qualitatively different among regions of Europe and Asia [53]. During the Holocene, steppe biomes occurred recurrently [54,55], coinciding with major dry events leading up to glacial conditions at different time intervals: ~ 11.000-9.500 years BP, ~ 8000-7000 years BP and 4000-3000 years BP [54,56]. More recently, an overall more arid period has been described in Eurasia during the last 4500 years (e.g. [39,57]). This, together with the increasing anthropogenic landscape transformations from 4000--3000 years ago (e.g. clearance of forested areas, cultivation, cattle grazing [49,58,59]), may have provided, either naturally or artificially, new steppe-like habitats for many species to colonize [60,61]. This temporal pattern is consistent with our data and could explain the recent population growths detected in both groups. The earlier and slower population growth detected in the SW group might have been associated with an increase in the extent of suitable open habitats after a major Holocene climate change dated around 8000 years BP [62]. In contrast, the existence of large steppe extensions in the East together with a lack of evidence supporting the aforementioned cooling event 8000 years ago in this region [62] would explain the lack of synchronous expansions of Montagu's harrier population groups. Additionally, studies on pollen spectra have clearly indicated that around 3000 and 1000-500 years BP steppe biomes were relatively abundant in the eastern part of the range (e.g. [54]), thus agreeing with the fast population growth observed in the northeastern populations of Montagu's harrier around that period of time (Figure 3).

## Conclusions

Our results point to a short isolation time, relatively recent population expansions and relaxed philopatry as the main factors determining the relative genetic homogeneity observed across populations of a steppe-associated raptor species. In contrast to the traditional view [3,63], our findings do not support an important role of southern Mediterranean peninsulas for extensive colonization of formerly treeless northern regions. In our case, rather than a source of postglacial colonization, the Iberian Peninsula would represent an area of postglacial refuge for steppe fauna. This finding implies that the population genetic models of glacial isolation and postglacial colonization developed for temperate taxa might have limited applications for steppe species. However, there is still little evidence for the direct effect of past climatic events on the genetic variability and phylogeographic structure in steppe-associated fauna at a regional or continental scale (but see [9,64]). Our study has added new insights into the knowledge of how genetic variation in steppe-associated taxa has been influenced by late Pleistocene and Holocene climatic changes. Future research should include a comparative approach, which would allow the comparison of phylogeographic patterns in a wider range of co-distributed species. This would contribute to a better understanding of how glacial cycles have sculpted the genetic variation of steppe-associated taxa in Eurasia.

## Methods

### Sampling

We analyzed genetic material from 284 Montagu's harrier specimens collected in 13 localities across the species' breeding range, from Spain to Kazakhstan, and from two wintering areas (Senegal, and Pakistan) (Table 1). Samples were grouped *a priori *according to sampling locality, and these groups were considered as populations for genetic analyses (see Table 1 and Figure 1). All samples are contemporary (collected in 1999-2009) and consisted of blood (n = 204) or feathers (n = 80). When nestlings were used as a source for DNA (<10% of samples) we used only one chick per brood to avoid pseudo-replication of mitochondrial haplotypes.

### DNA isolation, polymerase chain reaction (PCR) and sequencing

Blood samples were digested (8 h) in 250 µL SET buffer in the presence of SDS (2%) and proteinase K (10 ng/µL). Feathers were processed like blood samples but increasing proteinase K (20 ng/µL) and time of digestion (16 h). Total genomic DNA was extracted using standard NH_4_Ac protocol. Purified DNA was diluted to a working concentration of 25 ng/µL. We amplified two mitochondrial regions including partial tRNA-Trp and NADH dehydrogenase subunit 2 (ND2) and a portion of the cytochrome oxidase subunit I (COI) via polymerase chain reaction (PCR). Primers L5216-H5766 and L5758-H6313 were used for the amplification of ND2 gene [65], and BirdF1 and BirdR1 [66] for the COI fragment. PCR reactions were run using the following parameters: denaturation at 95 °C for 3 min, followed by 35 cycles of 94 °C for 60 s, 54 °C for 60 s, and 72 °C for 60 s, and a final extension at 72ºC for 5 min. PCRs contained approximately 25 ng of template DNA, 1× PCR buffer (Biotools), 0.25 mM of each dNTP, 0.3 µM of each primer, 2 mM MgCl_2_, and 0.5 U of *Taq *DNA polymerase (Biotools) in a total volume of 10 µL.

PCR-products were purified with Exonuclease I and Shrimp Alkaline Phosphatase enzymatic reactions (United States Biochemical). Purified reactions were sequenced in an ABI 3130 automated sequencer (Applied Biosystems) using dye-terminator chemistry (BigDye kit 3.1, Applied Biosystems) with the same primers used for PCR. All sequences are accessible at GenBank (Additional File 1).

### Genetic diversity and population structure analyses

We edited and aligned DNA sequences using Bioedit [67] and Clustal W [68]. Arlequin 3.5.1.2 [69] was used to determine the number of haplotypes and variable sites, and to calculate genetic diversity in each population (at the haplotype and nucleotide levels). Genetic differentiation between populations was tested using pairwise Φ_ST _comparisons for each mtDNA region and for the concatenated data set, using a Tamura-Nei evolutionary model [70], as this is the closest model to the one inferred for our data set in Modeltest 3.7 [71]. The significance of pairwise Φ_ST _comparisons was given by a *P *value calculated using 10,000 random permutation tests; p-values were further adjusted according to sequential Bonferroni corrections for multiple tests [72]. Evidence for population genetic structure was assessed using an analysis of molecular variance (AMOVA) as implemented in Arlequin 3.5.1.2. Tamura-Nei distances plus gamma correction (α = 0.2487) were selected for the concatenated data set. We first examined overall differences among populations (i.e. sampling localities without grouping) and then we determined the contributions of different grouping scenarios to the partitioning of genetic variation in the dataset. For this purpose, we tested three hypothetical scenarios (Table 2): (1) differentiation explained by the species current geographic distribution. We compared south-western (SW) vs. north-eastern populations (NE), which represent currently samples from a large and continuous breeding area (SW) and smaller populations with a more overall patchy distribution, both separated by a gap in the overall breeding distribution range ([73]; see Figure 1); (2) differentiation between populations separated by geographic barriers (i.e. mountain ranges: Pyrenees/Alps/Urals) as potential barriers to gene flow. For this, we compared Spanish, Western-central European, Eastern European, and Asian populations; (3) differentiation in relation to the intercontinental migratory divide (i.e. differences between populations wintering in Africa vs. those wintering in the Indian subcontinent).

Since all Montagu's harrier overwintering in the Indian subcontinent come from Asian breeding populations [27], winter samples from the Indian sub-continent (Pakistan) were included into the NE population group (in scenario 1) or into the Asian population group (in scenarios 2 and 3). Likewise, in scenario 3, samples from Senegal were pooled into the group of Montagu's harrier populations wintering in Africa. In all cases, we repeated these analyses without including samples from the two overwinter sites.

A pattern of isolation-by-distance was explicitly tested using Mantel tests to compare pairwise geographic and genetic distances between populations. These were statistically tested using linearized pairwise differentiation indexes (Φ_ST _/(1-Φ_ST_) in Arlequin 3.5.1.2. The statistical significance of correlations between distance matrices was obtained from 5,000 random permutations of matrix elements.

### Phylogenetic analysis

Phylogenetic relationships of Montagu's harrier were reconstructed by examining mtDNA sequence variation in all samples. The best-fit evolutionary model for the concatenated data set was determined using the Akaike information criterion implemented in Modeltest 3.7. A Maximum-Likelihood (ML) tree was built using a heuristic search starting from a neighbour-joining tree and a tree bisection reconnection (TBR) algorithm for branch swapping, with random addition of sequences in PAUP* 4.0 [74]. The statistical support for internal branches of the tree was estimated by 1,000 bootstrap replicates. This model was also used to carry out Bayesian inference (BI) of phylogeny as implemented in MrBayes v3.1.2 [75], simulating four simultaneous Monte Carlo Markov Chains (MCMC) for 5 × 10^6 ^generations each. The first 250,000 generations were discarded as burn-in. Bayesian posterior probabilities were obtained to assess the robustness of the BI trees. Trees were rooted with one sequence of *C. macrourus*, which was used as outgroup (Additional File 1).

We also represented the genealogical relationships of all the analyzed samples of *C. pygargus *with a haplotype network calculated using the median-joining algorithm [76] in NETWORK 4.5 http://www.fluxus-engineering.com.

### Demographic history

Signatures of demographic changes or selection in the recent history of *C. pygargus*, considering a model of mutation-drift equilibrium, were addressed using analyses based on different coalescent approaches. Firstly, we tested the data against a neutral Wright-Fisher model using Fu's *Fs *[77] and Tajima's *D *[78], which aim to identify an excess of recent single nucleotide substitution caused by population growth, bottleneck, or background selection. We performed this test in Arlequin 3.5.1.2. Significance of the statistics was determined by 1000 coalescent simulations of the neutral model, where *P *must be less than 0.02 to be significant due to the non-normal distribution of the *Fs *statistic [77]. Secondly, because departures from neutrality are often caused by changes in effective population size, we generated a Bayesian Skyline Plot (BSP) to explore changes in genetic diversity occurring at a certain time period within a given genealogy using MCMC based sampling, and to generate the posterior distribution of the effective population size at that time (*N_e_*) [79]. We used a strict molecular clock and a range of substitution rates estimated for other bird species (0.02-0.055 substitutions/site/Myr;; [13,80,81]. Four independent analyses were performed in BEAST v1.5.4 [79] and ran for 4 × 10^7 ^generations with a sampling frequency of 1000 steps. Convergence was assessed using Tracer v1.5 [82] and uncertainty in parameter estimates reflected in values of the 95% highest posterior density (HPD). We also used BEAST v1.5.4 to estimate the time to the most recent common ancestor (TMRCA) for each group of sequences analyzed as well as for the complete data set.

Thirdly, because AMOVA tests revealed genetic breaks between geographical regions (SW vs. NE, scenario 1), we used the program IMa [83] to test the hypothesis of a shared-history scenario of isolation with migration for the SW and NE population groups. This model assumes that an ancestral population of constant size and population parameter θ_A _separated into two populations (SW and NE) at time *T*, to simultaneously determine (1) time since divergence (*t*), (2) effective population sizes of each population (θ*_SW _*and θ*_NE_*) and the ancestral population (θ_A_) at time of split, and (3) immigration rates (*m_SW _*and *m_NE_*). We ran three replicate runs with a random seed to initiate each run. In all analyses, we used at least 20 Markov-coupled chains with a geometric heating scheme, a burn-in of 200,000 steps, and run until the effective sample sizes (ESS; see [83]) for each parameter were at least 500. To ensure proper chain mixing and parameter convergence, all parameter trend lines were visually inspected and three independent runs, which differed only in starting random seed, were compared. To convert IMa parameter estimates to biologically meaningful values, the parameters were scaled to a substitution rate of *µ *= 4 × 10^-8 ^substitutions per site per year (s/s/y). We also used a lower (*µ *= 2 × 10^-8 ^s/s/y) and an upper (*µ *= 5.5 × 10^-8 ^s/s/y) limit for this conversion, as indicated for the BSP analysis. To estimate generation time (*g*) we used the equation *g *= *α *+ (*s*/(1- *s*)), where *α *is the age of first reproduction in females and *s *is the expected adult survival rate [84]. Age at which Montagu's harrier females reach maturity was set at 2 years and estimated adult survival rate at 0.67, according to published data [24,85]. Therefore, we considered a generation time (*g*) of 4 years to express the output parameters (θ*_SW_*, θ*_NW_*, θ_A_, *m_SW_, m_NW_*, *t*) in demographic units as follows: effective population size: *N *= θ/4µ*g*; number of migrants per generation: *M *= θ × *m*/2*g*; divergence time in years: *T *= t*g*/2µ.

## Authors' contributions

JTG conceived and designed the study, collected samples, helped in molecular genetic work, performed analysis and drafted the manuscript. FA participated in the study design, carried out molecular genetic work, performed analysis and helped to draft the manuscript. FM, JT, AS, VB participated in the study design, collected samples and helped to draft the manuscript. BA conceived and designed the study, collected samples and helped to draft the manuscript. All authors read and approved the final manuscript.
